# THE DYNAMIC AND RECIPROCAL RELATIONSHIP BETWEEN PERCEIVED DISCRIMINATION AND COGNITIVE FUNCTION IN LATER LIFE

**DOI:** 10.1093/geroni/igad104.3116

**Published:** 2023-12-21

**Authors:** Takashi Amano, Yuane Jia, Audrey Redding-Raines

**Affiliations:** Rutgers University, Newark, New Jersey, United States; Rutgers University, Newark, New Jersey, United States; Rutgers University-Newark, Newark, New Jersey, United States

## Abstract

Studies have suggested that perceived discrimination is a potentially modifiable risk factor for cognitive health in later life. However, there is a lack of focus in the literature on the changes and directionality of the relationship between these two constructs. This study, based on socioemotional selectivity theory, investigates the dynamic and reciprocal relationship between perceived discrimination and cognitive function in later life. Data were drawn from four waves of the Health and Retirement Study (HRS; 2006, 2010, 2014, and 2018). We utilized a subsample of respondents who completed the psychosocial and lifestyle questionnaire in at least three waves. A total of 4,101 people who were 51 and older were included. Cognitive function was measured by the abbreviated version of the telephone interview for cognitive status (TICS-27). Perceived discrimination was measured using scores of the perceived everyday discrimination scale. Random intercept cross-lagged panel model (RI-CLPM) was utilized. Age was associated with lower perceived discrimination (b=-0.224, p<.0001). While lower cognitive function was cross-sectionally associated with higher perceived discrimination in all racial/ethnic groups, higher everyday discrimination predicted subsequent lower cognitive function only among White respondents. The model fit statistics were favorable: CFI=0.968; SRMR=0.045. Results suggested that a decline in cognitive function may exacerbate perceived discrimination which may result in further reduction of cognitive function in general. Lifetime experience of discrimination was discussed as a possible source of the racial/ethnic variations in the relationship. Further study is needed to examine whether this relationship holds among people with cognitive impairment or dementia.

